# Qin-Qiao-Xiao-Du formula alleviate influenza virus infectious pneumonia through regulation gut microbiota and metabolomics

**DOI:** 10.3389/fmed.2022.1032127

**Published:** 2022-10-14

**Authors:** Bo Lian, Shasha He, Hui Jiang, Yuhong Guo, Xuran Cui, Tao Jiang, Rui Su, Yuehong Chen, Chunxia Zhao, Mina Zhang, Yahui Hu, Haoran Ye, Jiaqi Ning, Xiaolong Xu, Qingquan Liu

**Affiliations:** ^1^Beijing Hospital of Traditional Chinese Medicine, Capital Medical University, Beijing, China; ^2^Beijing Institute of Chinese Medicine, Beijing, China; ^3^Beijing Key Laboratory of Basic Research with Traditional Chinese Medicine on Infectious Diseases, Beijing, China; ^4^Department of Traditional Chinese Medicine, Beijing Chao-Yang Hospital, Capital Medical University, Beijing, China; ^5^Beijing Chest Hospital, Capital Medical University, Beijing, China; ^6^State Key Laboratory of Pathogen and Biosecurity, Beijing Institute of Microbiology and Epidemiology, Academy of Military Medical Sciences, Beijing, China; ^7^Dongzhimen Hospital Affiliated to Beijing University of Chinese Medicine, Beijing University of Chinese Medicine, Beijing, China; ^8^Tianjin University of Traditional Chinese Medicine, Tianjin, China

**Keywords:** influenza virus, Traditional Chinese medicine, pneumonia, gut microbiota, metabolism

## Abstract

Qin-Qiao-Xiao-Du (QQXD), a traditional Chinese medicine (TCM) formula, has been used in the clinical treatment of influenza virus pneumonia. However, the effects and mechanisms of QQXD on influenza virus pneumonia remain unknown. Therefore, this study explores the mechanisms of QQXD in the treatment of influenza virus pneumonia from the point of view of intestinal flora and metabolism. The results showed that QQXD was able to reduce mortality, weight loss, lung viral load, lung index, and lung injury in influenza virus mice. A cytokine array found that the QQXD attenuated the expression of serum IL-1α, IL-4, IL-12(P70), and TNF-α. Subsequently, 16s rRNA gene sequencing showed that QQXD could increase the relative abundances of Gemmiger, Anaerofustis, Adlercreutzia, and Streptococcus and decrease those of Dehalobacteriu, Burkholderia, Prevotella, Butyrimimonas, Delftia, and others. Meanwhile, targeted metabolic profiling analysis showed that QQXD could regulate nitrogen metabolism, phenylalanine metabolism, valine, leucine, and isoleucine biosynthesis. Correlation analysis demonstrated that the regulatory effect of QQXD on the cyanoamino acid metabolism pathway was associated with changes in the abundance of Parabacteroides, Pediococcus, and Clostridium in influenza mice. In conclusion, our study revealed that QQXD can inhibit influenza virus replication, suppress cytokine storms, and protect mice from influenza virus infection pneumonia. The mechanisms are likely to be related to improved gut microbiota dysbiosis, increased intestinal carbohydrate metabolism, and up-regulated cyanoamino acid metabolism pathways.

## Introduction

Influenza is an acute respiratory infectious disease caused by the influenza virus and is characterized by fever, cough, headache, muscle and joint pain, severe malaise, sore throat, and a runny nose. Globally, these annual epidemics are estimated to result in approximately 3–5 million cases of severe illness, and approximately 290,000–650,000 respiratory deaths (1). Influenza pandemic causes considerable incidence rate and mortality each year, and may have the same impact as COVID-19 (2). Vaccines are the most effective way to prevent influenza (3). However, due to the antigenic drift of influenza viruses, the preventive effect of influenza vaccines on influenza viruses is weakened (4). Influenza vaccines have limited preventive effects. Influenza is a common epidemic infectious disease in the world, except for the COVID-19. As COVID-19 continues to spread around the world, it will overlap with influenza virus infections in each upcoming influenza season, thus placing a significant burden on people’s health (5). Therefore, given the annual incidence of influenza and the preventive effects of vaccines, it is important to develop effective therapies or supplements to effectively inhibit influenza viruses.

Influenza viruses can cause hemorrhagic bronchitis, diffuse alveolar injury, and pneumonia within a few hours (6). Pneumonia is a common complication of influenza virus infection. It is highly likely to develop into severe influenza if treatment is not timely or appropriate, and influenza virus pneumonia is a leading cause of death (7). Influenza virus pneumonia is also easily associated with bacterial and fungal infections (8, 9). After influenza virus infection, inflammatory factors can not only promote viral clearance, but also further cause lung tissue damage (10–12). Influenza virus infection of the host causes an imbalance in the intestinal flora, which is more likely to lead to bacterial infection (13–15). After the virus infects the host, it undergoes substantial and intimate interactions with the symbiotic microbes in the host. Numerous lines of evidence show that the symbiotic microbiota regulates viral infection through a variety of mechanisms (16). In addition, intestinal microbiota can affect the host metabolism, which may contribute to the progression of viral infection. A study has demonstrated that HIV infection drastically alters the microbial community, with the species responsible for the metabolism of four amino acids (17). A meta-analysis found that Traditional Chinese medicine (TCM) in the treatment of influenza reduced the fever time and improved the total effective rate (18). One study has concluded that TCM can inhibit influenza virus replication, prevent the apoptosis of tissue cells induced by influenza virus, reduce the oxidative stress damage caused by influenza virus, and regulate immunity (19). TCM can regulate the intestinal flora of the host, including regulating the composition of intestinal microbiota and regulating its metabolism. At the same time, intestinal microbiota can also transform compounds with low oral TCM utilization and reduce TCM toxicity, suggesting that intestinal microbiota may be key to explaining TCM treatment mechanisms (20–24).

Qin-Qiao-Xiao-Du formula (QQXD) is a clinically effective prescription designed for the treatment of influenza virus pneumonia by Professor Qingquan Liu, a leading researcher in the therapy of infectious diseases using TCM. The selection and compatibility rules for decoction tablets are in line with the well-known prescriptions of classical TCM for the treatment of epidemic diseases (the results have not been published). The herbs used in QQXD have a certain inhibitory effect on influenza viruses and other viruses, reducing the inflammatory reaction, and can also reduce lung injury (25–29). However, the effects and mechanisms of QQXD on influenza virus pneumonia are still unclear. In this study, we first identified the main bioactive compounds in plasma after oral administration of QQXD by ultra-performance liquid chromatography-tandem mass spectrometry (UPLC-MS). The efficacy of QXD on influenza mice was then measured by influenza virus replication, inflammatory response, and lung injury. In addition, we investigated the mechanisms of QXD in the treatment of influenza viral pneumonia using 16S rRNA gene sequencing and targeted metabolic profiling.

## Materials and methods

### Preparation of Qin-Qiao-Xiao-Du formula

The five herb ingredients of QQXD were purchased from Beijing Hospital of Traditional Chinese Medicine (Beijing, China). The decoction pieces were stored in Beijing Institute of Traditional Chinese Medicine (Beijing, China) and kept by a specially assigned person. The composition and dosage of QQXD include *Scutellaria baicalensis Georgi* (Lamiaceae; *Scutellariae radix* 30 g), *Forsythia suspensa (Thunb.) Vahl* (Oleaceae; *Forsythia fructus* 30 g), *Mentha canadensis* L. (Labiatae; *Menthae haolocalycis herba* 15 g), *Platycodon grandiflorus (Jacq.) A.DC.* (Platycodonaceae; *Platycodonis radix* 30 g), and *Glycyrrhiza glabra L.* (Leguminosae; *Glycyrrhizae radix et rhizome* 15 g). Soak the decoction pieces except *Mentha canadensis L.* in 7 times the volume of pure water for 30 min, decoct twice for 45 min each, add *Mentha canadensis L.* after concentration and decoct to prepare 2 g/ml medicinal liquid.

For the dose conversion between drugs in mice and human drugs, refer to the Experimental methodology of pharmacology (30) and convert according to the body surface area estimation method. The conversion factor is 9.1. The equivalent dose is set as the medium dose, the low dose is 0.5 times of the equivalent dose, and the high dose is 2 times of the equivalent dose.

### Ultra-performance liquid chromatography-tandem mass spectrometry of Qin-Qiao-Xiao-Du formula

Plasma samples were collected at 30, 60, and 90 min after oral administration of QQXD decoction for 5 days. A system (Waters Corp., Milford, MA, United States) equipped with a HESI-II probe was used to identify the main components of QQXD, as previously reported (31). The positive and negative HESI-II voltages were set to 3.5 and –3.0 kV, respectively, and the vaporizer temperature was set to 400^°^C. Both sheath gas and auxiliary gas are nitrogen, and collision gas is high-purity nitrogen. The mobile phase was composed of A (acetonitrile) and B (0.1% (v/v) formic acid aqueous solution). The HPLC elution conditions were optimized as follows: 0–5 min: 5–10% A; 5–12 min: 10–15% A; 12–20 min: 15–30% A; 20–30 min: 30–95% A; 30–32 min: 95% A; 32–32.1 min 95–5% A; and 32–36 min: 5% A. The flow rate and the column temperature were set to 0.35 ml/min and 35^°^C, respectively. Data were collected and processed by Xcalibur 4.2 software (Waters Corp.).

### Animals, virus and grouping

All experimental procedures performed on the animals were conducted under the National Institutes of Health Guidelines on Laboratory Research. The experiment was conducted under the supervision and evaluation of the ethics committee of the Experimental Animal Center of the Academy of Military Medical Sciences (permit number: 2020-061).

192 specific-pathogen-free BALB/C female mice weighing 16 g ± 1 g were purchased from Beijing Vital River Laboratory Animal Technology Co., Ltd, (Beijing, China). The mice were placed in a 12 h light/dark cycle, at a relative temperature of (23^°^C ± 1^°^C) and relative humidity (50% ± 5%), and could freely contact with food and water. The mice were fed adaptively for 1 week.

The Influenza A virus [A/California/07/2009(H1N1), CA07] used in this experiment was provided by Department of Virology (State Key Laboratory of Pathogen and Biosecurity, Beijing Institute of Microbiology and Epidemiology, China). The titer of influenza virus was 1.4*10^7^pfu/ml, LD50 was 7*101.5 pfu, and stored in a –80^°^C refrigerator.

Mice were randomly divided into the following six groups: Group A: control group (Control); Group B: influenza virus-infected group (Model, 221 pfu); Group C: influenza virus-infected and Oseltamivir treatment group as positive group (Oseltamivir, 221 pfu, 100 mg/kg/d); Group D: influenza virus-infected and low dose QQXD treatment group (QQXD-L, 221 pfu, 9.1 g/kg/d); Group E: influenza virus-infected and medium dose QQXD treatment group (QQXD-M, 221 pfu, 18.2 g/kg/d); Group F: influenza virus-infected and high dose QQXD treatment group (QQXD-H, 221 pfu, 36.4 g/kg/d).

### Modeling and treatment

Mice were fully anaesthetized using inhalation of diethyl ether and infected through the intranasal application of 1 × LD50 influenza virus suspension. This caused upper and lower respiratory tract infections in the mice. Control group inhaled phosphate buffered saline (PBS). Gavage was performed 1 h after infection with influenza virus. Control and Model groups were given PBS for 5 days. Oseltamivir and QQXD groups were given corresponding drugs for 5 days. All the mice were euthanized after 5 days of treatment for the infection. Blood, lung and intestinal contents were collected.

### Survival experiment

The mice were randomly divided into six groups, including Control (*n* = 11), Model (*n* = 16), Oseltamivir (*n* = 16), QQXD-L (*n* = 16), QQXD-M (*n* = 16), QQXD-H (*n* = 16). Mice were infected with the virus with diluted influenza virus (1 × LD50) through the nose, and normal mice were infected with an equal volume of PBS in the same way. All groups were monitored for 8 days after infection. The weight and number of mice were recorded daily. Life spans and survival rates were calculated.

### Lung index and histology

Lung samples were weighed and calculated according to the following formula: lung index = (mouse lung weight/mouse weight) × 100%. The right lung tissue was fixed with 4% paraformaldehyde and modified into a square block of approximately 0.2 cm^3^ with a blade. Dehydrate and embed the tissue block and cut it into a 0.5 μm slice for dyeing. The size of the microscopic field employed in the analysis was × 200. Pathologists assessed damage to lung tissue in all sections.

### Lung viral load

Total RNA was extracted from the right lung homogenate of mice using a Pure Link viral RNA/DNA Mini Kit (Thermo Fisher Scientific, New York, NY, USA; 12280-050). Real-time PCR was performed using a One Step TB Green Prime Script PLUS RT-PCR Kit (TaKaRa; RR096A) and a Light Cycler 480 II (Roche, Mannheim, Germany).

### Inflammatory factor

The Bio-Plex Pro Mouse Cytokine Grp I Panel 23-plex Kit (Bio-Rad; #M60009RDPD) was used to determine the serum inflammatory factors in mice, including IL-1α, IL-4, IL-6 and TNF- α.

### Gastrointestinal microbiota

Total DNA from the mouse gut was extracted, the DNA was quantified using NanoDrop, and the quality of the extracted DNA was detected using 1.2% agarose gel electrophoresis. DNA samples were used as templates for PCR amplification of the V3-V4 region of the bacterial 16S rRNA gene. Quant-iT PicoGreen dsDNA Assay Kit (Thermo Fisher Scientific; P7589) was used to quantify the PCR products recovered from PCR amplification. Based on the fluorescence quantification results, each sample was mixed according to the corresponding proportion of the required sequencing volume for each sample.

The sequencing library was prepared with TruSeq Nano DNA LT Library Prep Kit (Illumina; 20015965). The quality inspection shall be carried out before starting the machine, and high-throughput sequencing shall be carried out after the quality inspection. On QIIME2 (2019.4) software, DADA2 was called for sequence denoising to obtain amplification sequence variants (ASVs) and operational taxonomic units (OTUs), annotated species classification and constructed phylogenetic tree. The samples were subjected to species composition analysis, alpha diversity analysis, beta diversity analysis, species difference analysis, and functional potential analysis.

### Metabolomics

Intestinal contents were added to pure water, homogenized with zirconium oxide beads, and then Methanol (Optima LC-MS) containing an internal standard was added to extract the metabolites. Following procedures were performed on an Eppendorf epMotion Workstation (Eppendorf Inc., Humburg, Germany), added the newly prepared derivatization reagent to the samples, sealed them at 30^°^C for derivatization for 1 h, then added ice-cold 50% methanol solution to dilute the samples, and stored it at –20^°^C for 20 min. The samples were centrifuged at 4^°^C and the supernatant was removed for analysis by liquid chromatography tandem mass spectrometry (LC-MS). The absolute concentrations of 228 metabolites were targeted measured using the UPLC-MS/MS system in 21 samples by Wayen Biotechnologies (Shanghai, China). Metabolic classification, principal component analysis, differential metabolite screening, and pathway enrichment analysis of mouse intestinal contents were analyzed.

### Correlation analysis of gastrointestinal microbiota and metabolomics

The genus level differential flora and differential metabolites (potential biomarkers) were imported into R software (R version 4.1.3) for visualization, and their relationship was displayed by a heat map.

### Statistical analysis

Statistical analysis was performed using Prism 8.0 (GraphPad Software, CA). Data are presented as means ± SEM. Significant differences between the two groups were evaluated by a two-tailed unpaired Student’s *t*-test. Significant differences in more than two groups were evaluated by a one-way analysis of variance (ANOVA) multiple comparisons test.

When analyzing gut microbiota sequencing data, a two-tailed Wilcoxon rank-sum test by R software (R version 4.1.3) was performed. LDA Effect Size (LEfSe) analysis by Python Project was performed. Random forest analysis was performed using the QIIME2 software. When analyzing metabolites, iMAP (v1.0, Metabo-Profile, Shanghai, China) was used for statistical analyses, including PCA and pathway analysis. When analyzing the relationship between flora and metabolism, the Spearman correlation analysis by R software (R version 4.1.3) was performed. The differences were considered to be significant at *p* < 0.05.

## Results

### Identification of main compounds of Qin-Qiao-Xiao-Du formula medicated serum

**FIGURE 1:**
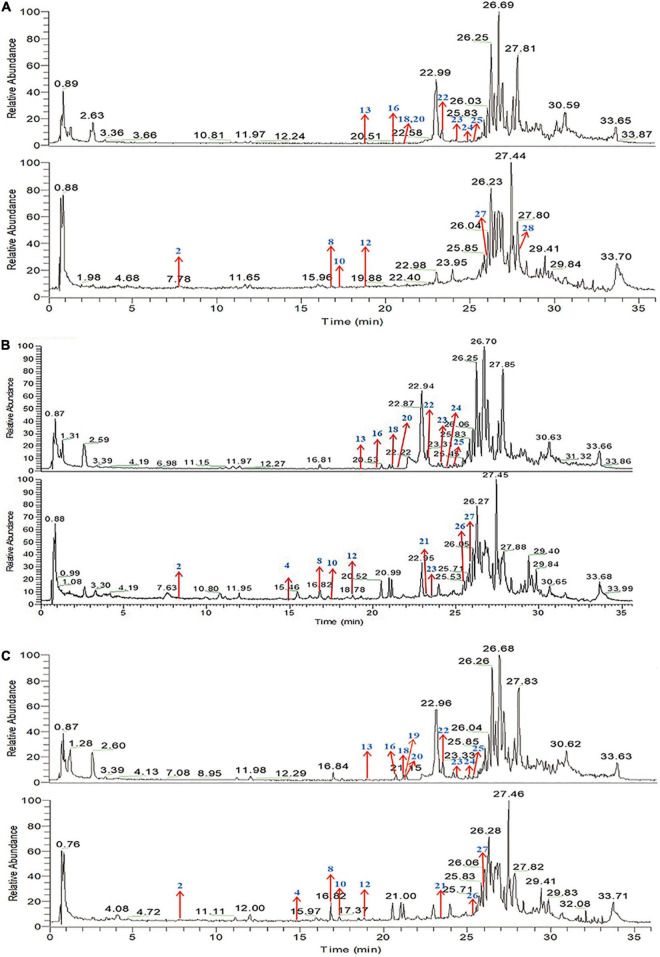
Total ion chromatogram (TIC) in positive ion mode and negative ion mode by UPLC-MS/MS. **(A)** Sample-30 min. **(B)** Sample-60 min. **(C)** Sample-90 min.

The main bioactive components of QQXD decoction were identified by UPLC-MS method after oral administration for 30, 60, and 90 min of oral administration. Total positive ion and negative ion chromatograms of samples illustrated the main bioactive compounds of QQXD medicated serum (Figure 1). Of these, eighteen are major compounds, including liquiritin, baicalin, platycodin D, baicalein, wogonoside, glycyrrhizic acid, and enoxolone, among others. The specific components of QQXD are shown in Supplementary Table 1.

### Effects of Qin-Qiao-Xiao-Du formula on weight, survival rate, lung viral load and lung index in influenza mice

**FIGURE 2:**
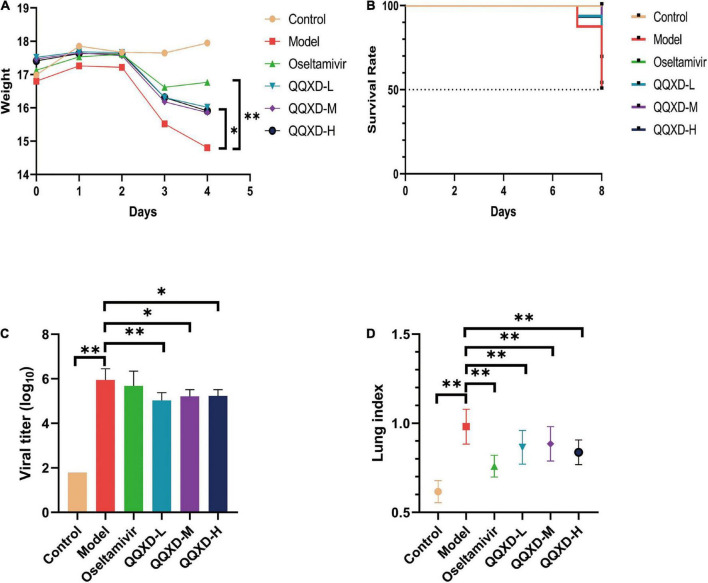
Effects of QQXD on weight loss, survival rate, lung viral load, and lung index in influenza mice. **(A)** Body weight changes. **(B)** Survival rate. **(C)** Lung viral load. **(D)** Lung index. *p < 0.05, ^**^p < 0.01.

The results showed that control mice did not lose weight. After influenza virus infection, Model, Oseltamivir, QQXD-L, QQXD-M, QQXD-H groups showed significant weight loss (Figure 2A). Influenza virus infection results in significant weight loss, which is controlled with Oseltamivir and QQXD.

No deaths have been reported with either Control or Oseltamivir in the survival trail. On the seventh day of influenza virus infection, Model reported two deaths, and QQXD-L and QQXD-H each reported one death. On the eighth day, Model reported six deaths, QQXD-L reported seven deaths, QQXD-M reported five deaths, and QQXD-H reported six deaths. One of themice in the QQXD-H died, and the cause of the drug and infection were ruled out as the cause. Oseltamivir and QQXD treated mice were protected from lethal infection. 50% of the mice in Model survived, while 68.75% and 53.33% of the mice in the QQXD-M and QQXD-L survived (Figure 2B).

We evaluated the inhibitory effect of QQXD on influenza viruses by using the lung viral loads from influenza mice. On the fifth day after the influenza virus infected the lung tissue of the mice, the virus massively replicated in the lung tissue. The lung viral load in Model increased significantly, while QQXD-L, QQXD-M, and QQXD-H significantly reduced the lung viral load (Figure 2C), and the difference was statistically significant. QQXD was able to reduce viral load in the lungs of mice.

The influenza virus infected the lung tissue of the mice and caused significant lung damage, including pulmonary edema and pulmonary hemorrhage, resulting in a significant increase in the weight of the mice’s lungs. We used lung index and hematoxylin-eosin staining of lung tissue to observe the effect of QQXD on lung injury in influenza mice. The lung index of mice in Model was significantly increased, while Oseltamivir, QQXD-L, QQXD-M, and QQXD-H significantly decreased the lung index of mice, and the difference was statistically significant (Figure 2D).

### Effects of Qin-Qiao-Xiao-Du formula on serum inflammatory factors and lung tissue

**FIGURE 3:**
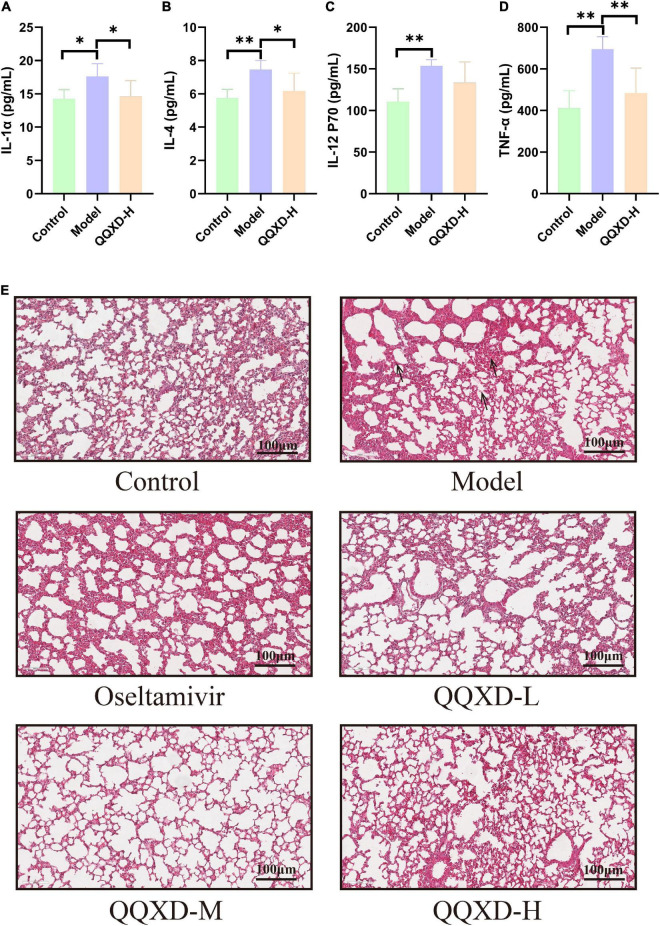
Effects of QQXD on inflammatory factors and lung histopathology in influenza mice. **(A)** IL-1α. **(B)** IL-4. **(C)** IL-12(P70). **(D)** TNF-α. **(E)** Hematoxylin-eosin staining of lung tissue; bar = 100 μm. *p < 0.05, **p < 0.01.

Influenza virus infection causes an inflammatory factor response. This study found that the inflammatory factors such as interleukin-1α (IL-1α), interleukin-4 (IL-4), interleukin-12(P70) [IL-12(P70)], and tumor necrosis factor-α (TNF-α) in Model group were significantly increased (Figures 3A–D), QQXD could significantly reduce IL-1α, IL-4, and TNF-α, and the difference was statistically significant (Figures 3A–D).

Hematoxylin and eosin (H&E) staining was used to observe the effect of QQXD treatment on the pathological changes of lung tissue in influenza mice (Figure 3E). In Control, lung damage occurred, the bronchi, perivascular spaces and interlobar septa were widened, and there was no obvious damage to the alveoli. In Model, the lung tissue was significantly damaged, the alveolar structure was destroyed, the bronchial epithelium was shed, the blood vessels in the tube wall were dilated and congested, there was inflammatory cell infiltration, and a large number of nuclear cells infiltrated in the alveolar wall. In QQXD, alveolar structure were damaged and bronchial epithelial leakage were also observed, but were less severe than those in Model.

### Effects of Qin-Qiao-Xiao-Du formula on the composition of intestinal flora in influenza mice

**FIGURE 4:**
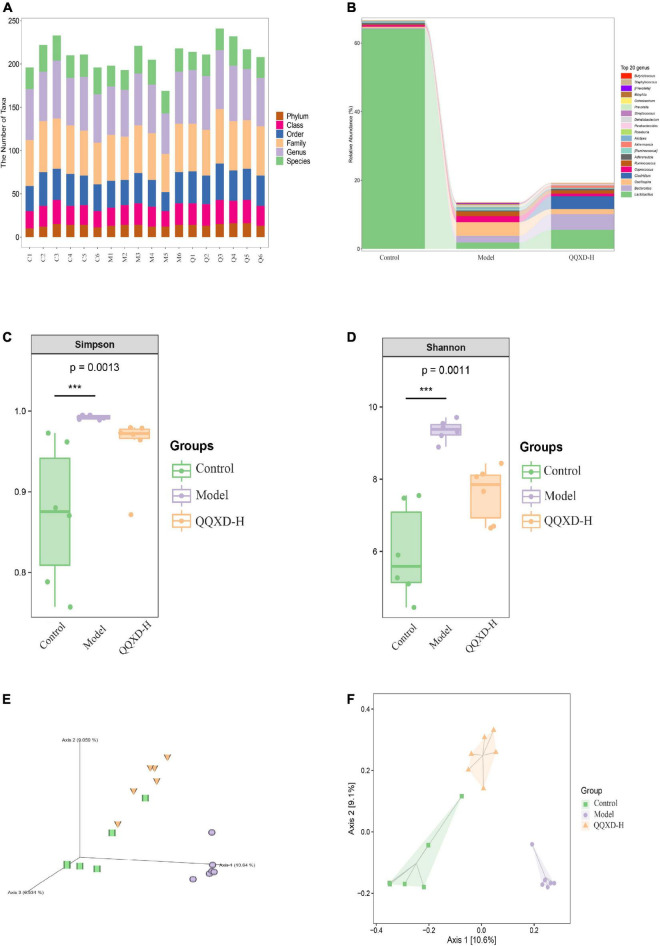
Composition and diversity of intestinal flora in influenza mice treated with QQXD. **(A)** Statistical chart of the number of microbial taxonomic units at each level. Control: C1–C6; Model: M1–M6; QQXD: Q1–Q6. **(B)** Genus level species composition histogram. **(C)** Simpson index. **(D)** Shannon index. **(E)** Jaccard based PCoA 3D. **(F)** Jaccard based PCoA with hull 2D. ***p < 0.01.

The DNA fragments of the communities were sequenced on the Illumina MiSeq/NovaSeq platform. The species were classified and annotated, and the differences in OTUs and taxonomic status identification results among different samples and groups were compared. The measured sample abundance was transformed by rarefaction to obtain the microbial community in each sample at each classification level (Figure 4A).

After normalizing the sample abundances within groups, the species richness at the genus level is analyzed. Control was mainly Lactobacillus, Model was mainly Lactobacillus and Oscillospira, and QQXD was mainly Lactobacillus and Bacteroides. Lactobacillus in Model and QQXD were significantly down-regulated (Figure 4B).

The Shannon Diversity Index takes into account both the richness and evenness of the community. The Simpson Index is a commonly used index to evaluate community diversity by calculating the probability that two randomly sampled individuals (Sequences) belong to different species (ASVs/OTUs). The higher the Simpson index, the more diverse the community. The Shannon index/Simpson index value is directly proportional to community diversity. This study found that the diversity of the intestinal community in Model was higher than those in Control and QQXD (Figures 4C,D). The Chao1 estimator and the Observed species richness are used to evaluate the richness of the community. The larger the Chao1 estimator and the Observed species richness, the higher the community richness. The Faith’s Phylogenetic Diversity indicates the relationship between the newly added ASV/OUT and another species in the community. The relationship between flora is proportional to the Faith’s PD index. The Pielou’s evenness index reflects community evenness, and Pielou’s evenness index is proportional to community evenness. The Good’s non-parametric Coverage estimator assesses the coverage of species in a community by sequencing, and the Good’s coverage index is inversely proportional to the fraction of undetected species. This study found that the richness of the intestinal community in Model was higher than those in Control and QQXD (Supplementary Figures 1A,B). The colonies in Model were far related and the intestinal community was more uniform, which was different from Control and QQXD (Supplementary Figures 1C,D). Control, Model and QQXD did not detect the low proportion of species (Supplementary Figure 1E). Principal coordinates analysis (PCoA) is used to reduce the dimension of multidimensional microbial data, and the main trend of data change is shown by the distribution of samples on the continuous sorting axis. The Jaccard distance shows the difference between different samples by counting the fraction of non-common species among all species in the sample. This study found that the intestinal community composition of Control, Model and QQXD were not similar in the corresponding dimensions, and there were differences among the three groups (Figures 4E,F).

### Screening of differential flora and analysis of functional pathways

**FIGURE 5:**
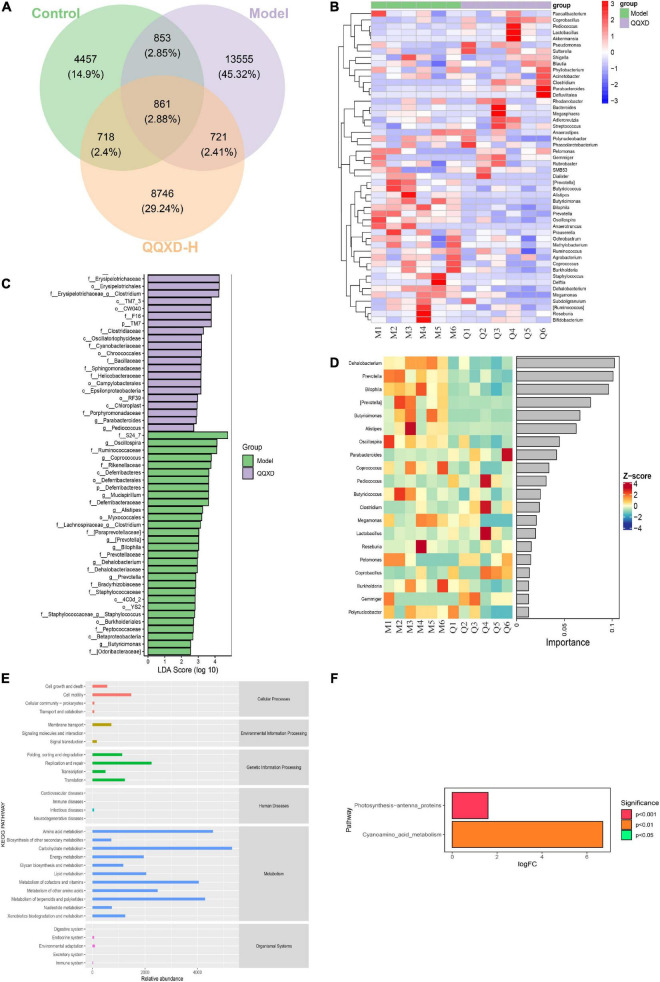
Analysis of differential flora and pathway enrichment. **(A)** Venn diagram of differential flora. **(B)** Genus level species composition cluster heat map. The red color block indicates that the abundance of this genus in this sample is higher than that in other samples, and the blue color block indicates that the abundance of this genus in this sample is lower than that in other samples. **(C)** Histogram of LDA effect values of marker species. **(D)** Top 20 important flora in genus level. **(E)** KEGG secondary function channel abundance diagram. **(F)** Difference between QQXD and Model KEGG metabolic pathway. A positive value of logfc on the horizontal axis indicates that QQXD is up-regulated [log2 (fold change)] relative to the Model, and a negative value indicates that it is down-regulated; the ordinates are labels of different KEGG metabolic pathways; Show the degree of significance in different colors.

In this study, the differences in ASV/OTU abundance among the three groups were compared. The results showed that there were 4,457, 13,555, and 8,746 unique ASV/OTU in Control, Model and QQXD, and 861 ASV/OTU in the three groups (Figure 5A). The abundances of flora in the three groups of samples were analyzed and found to be higher in Model. The abundance data for the top 50 genera in the average abundance were used to draw the heatmap. The results showed that in Model, Dehalobacteriu, Burkholderia, Prevotella, Butyricimonas, Delftia, Ochrobactrum, [Prevotella], Oscillospira, Butyricicoccus, Roseburia, Bifidobacterium, Coprococcus, Methylobacterium, Bilophila, Prauserella, [Ruminococcus], Polynucleobacter, Alistipes, Agrobacterium, Phyllobacterium, Staphylococcus, Megamonas increased in abundance, but decreased in Control and QQXD. The abundance of Gemmiger, Anaerofustis, Adlercreutzia, Streptococcus decreased in Model, and increased in Control and QQXD (Figure 5B). Lefse analysis was performed to find robust differential species among the three groups. In this study, linear discriminant analysis (LDA) was set to be greater than 2, *p* < 0.05. QQXD was Erysipelotrichi, TM7_3, CW040, etc. Model was S24_7, Oscillospira, Ruminococcaceae, etc. (Figure 5C). In this study, we applied random forest analysis to Model and QQXD differential flora, and obtained important differential flora mainly including Dehalobacterium, Prevotella, Bilophila, etc. (Figure 5D).

Phylogenetic Investigation of Communities by Reconstruction of Unobserved States (Picrust2) software obtained the abundance data of three groups of metabolic pathways and analyzed KEGG metabolic pathways. The results showed that the three groups of sample bacteria were mainly in transport and catabolism, membrane transport, signal transduction, replication and repair, infectious diseases, xenobiotics biodegradation and metabolism, and endocrine system are significantly enriched (Figure 5E). In this study, metabolic pathways with significant differences between groups were investigated after obtaining abundance data for the metabolic pathways. Compared with the model group, QQXD up-regulated cyanoamino acid metabolism and photosynthesis antenna proteins (Figure 5F).

### Effects of Qin-Qiao-Xiao-Du formula on the species and abundance of metabolites in influenza mice

**FIGURE 6:**
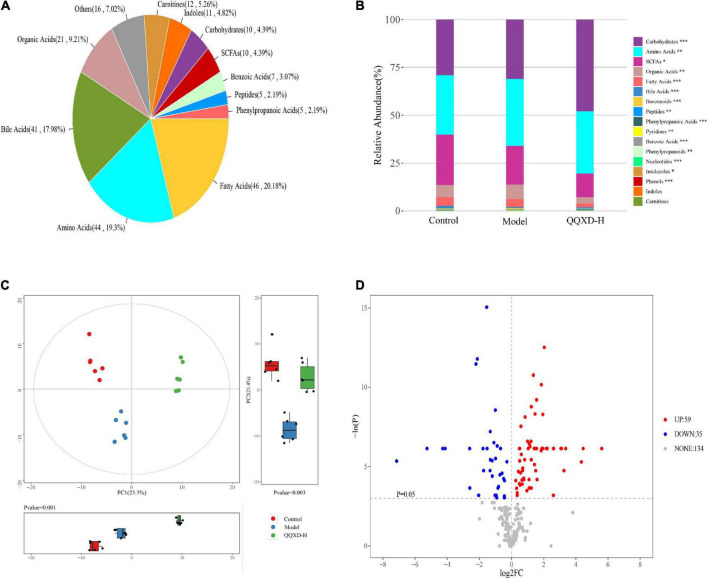
Metabolite composition, component analysis and inter-group differential metabolism. **(A)** Statistical pie chart of category and constituent ratio of metabolites. **(B)** Statistical stacked histogram of relative abundance of median values of three groups of various metabolites. **(C)** Three groups of PCA score diagram and corresponding principal component box diagram. **(D)** Comparison between QQXD and Model single dimensional metabolite volcano map. Threshold setting *p* < 0.05 and | log2fc | ≥ 0 (FC, fold change).

The absolute concentrations of 228 metabolites were targeted measured using the UPLC-MS/MS system in 21 samples, mainly including fatty acids, amino acids, bile acids and organic acids (Figure 6A). The average fractionation of metabolites in all samples was analyzed, and it was found that carbohydrates, amino acids and short-chain fatty acids (SCFAs) dominated. The metabolism of SCFAs in the QQXD and Model decreased significantly, the carbohydrate metabolism in QQXD increased, while the organic acids and fatty acids in Model increased (Figure 6B).

Multidimensional statistical analysis was used to analyze three groups of principal components, and principal component analysis (PCA) was used. The results showed that there were differences among the three groups of principal components (Figure 6C). A total of 94 differential metabolites were obtained by one-dimensional statistical analysis between Model and QQXD (Figure 6D).

### Screening differential metabolites and analysis of metabolic pathways

**FIGURE 7:**
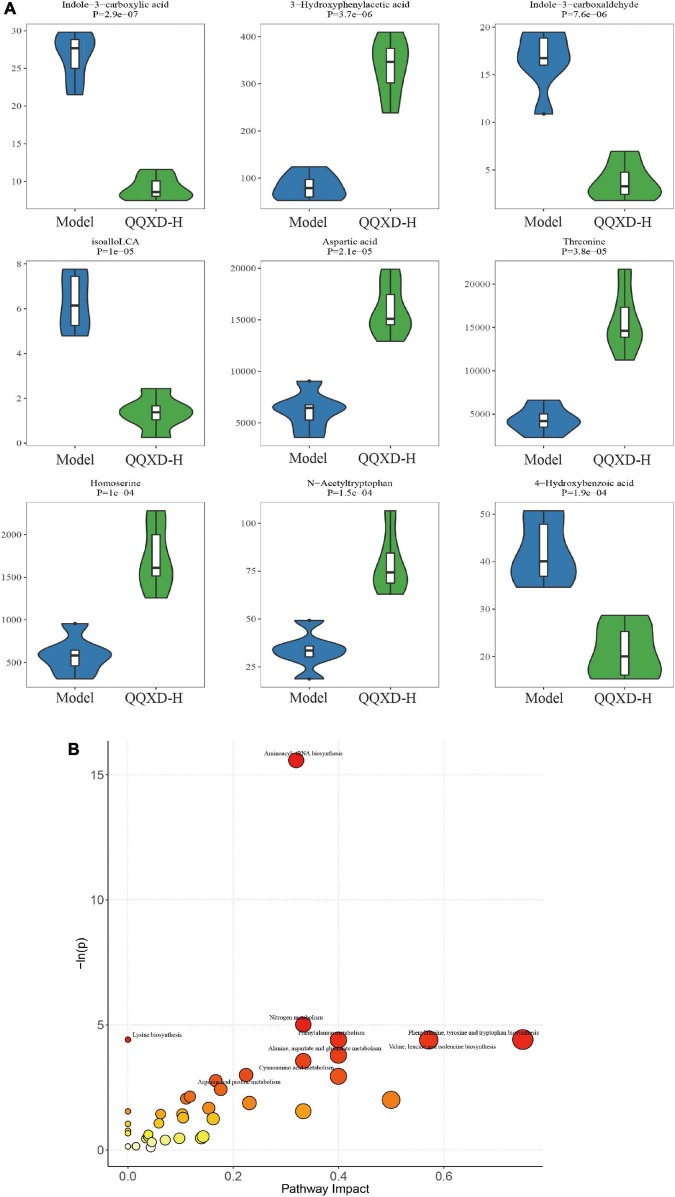
Differential metabolites and pathway analysis. **(A)** Violin diagram of differential metabolites. **(B)** Path analysis bubble chart.

In this study, 94 of the most significant differential metabolites were selected. In QQXD comparison Model, indole-3-carboxyacid, indole-3-carboxaldehyde, isoalloLCA, and 4-hydroxybenzoic acid decreased (Figure 7A), 3-hydroxyphenylacetic acid, aspartic acid, threonine, homoserine, and n-acetyltryptophan increased (Figure 7A). Control and QQXD’s single-dimensional and multi-dimensional acquisition of differential metabolites were intersected to select potential biomarkers with biological significance. Screening criteria: single-dimensional analysis *p* < 0.05, |log2FC| ≥ 0 and multi-dimensional analysis VIP > 1, a total of 93 potential biomarkers were obtained, and the selected mouse KEGG database was used for pathway analysis. The results showed that there were differences in Aminoacyl-tRNA biosynthesis, Nitrogen metabolism, Lysine biosynthesis, Phenylalanine, tyrosine and tryptophan biosynthesis, Phenylalanine metabolism, Valine, leucine, and isoleucine biosynthesis (Figure 7B).

### Correlation analysis of targeted metabolomics and gut microbiota

**FIGURE 8:**
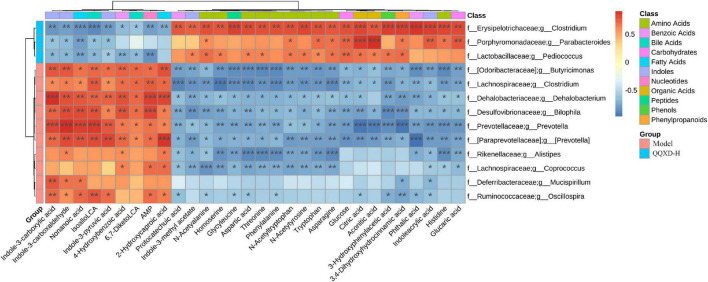
The heat map of correlation analysis between gut microbiota and targeted metabolic profiling. *p <0.05, **p <0.01, ***p <0.001.

13 species of genus-level differential flora and 30 different metabolites were conducted in the Spearman correlation analysis to identify the effect of QQXD on intestinal flora and the metabolic relationship of influenza mice (Figure 8). Strong correlations (*p* < 0.05) were observed in 307 microbes-metabolites pairs. We found that QQXD treatment of influenza mice increased the abundance of Parabacteroides, Pediococcus and Clostridium, affected the decreased metabolites Indole-3-carboxylic acid, Indole-3-carboxaldehyde, isoalloLCA, 4-Hydroxybenzoic acid, Indole-3-pyruvic acid, 6,7-DiketoLCA, Nonanoic acid, AMP and 2-Hydroxycaproic acid, and increased metabolites 3-Hydroxyphenylacetic acid, Aspartic acid, Threonine, Homoserine, N-Acetyltryptophan, Glucose, Citric acid, Indoleacrylic acid, Phthalic acid, Aconitic acid, Protocatechuic acid, Tryptophan, Phenylalanine, Histidine, 3,4-Dihydroxyhydrocinnamic acid, Asparagine, Glycyleucine, Glucaric acid, N-Acetyalanine, N-Acetytyrosine and Indole-3-methyl acetate. Prevotella, Butyricimonas, Mucispirillum, Dehalobacterium, Bilophila, and Clostridium had effects on the metabolism of influenza mice.

## Discussion

In this study, we identified 18 major active compounds in plasma after oral administration of QQXD decoction, including liquitin, baicalin, wogonoside, platycodin D, baicalein, glycorrhizac acid, wogonin, enolone, phillyrin, and so on. Then, we demonstrated that QQXD improved the survival outcomes, inhibited influenza virus replication, inhibited cytokine storms, and protected against lung injury in influenza mice. The mechanisms are likely to be related to improved gut microbiota dysbiosis, increased intestinal carbohydrate metabolism, and up-regulated cyanoamino acid metabolism pathway. To our knowledge, this is the first report to elucidate the protective effect and mechanism of QQXD on influenza virus pneumonia.

Influenza viruses adsorbed on the surface of the nasal cavity or oral mucosa and enter the human body. Within 48 hours, the newly replicated influenza virus reaches its peak (32). Influenza-related symptoms begin to appear 1–4 days after infection. For most infected people, influenza acute symptoms last 7–10 days (33). Inflammatory reactions occur simultaneously with the replication of the influenza viruses and the emergence of influenza symptoms. Influenza virus infection can be divided into three stages: innate immunity stage, autoimmunity stage and late virus clearance stage, which includes tissue regeneration and repair (34). After influenza virus infects epithelial cells, endothelial cells and alveolar macrophages produce the first wave of inflammatory factors, and then adaptive immune cells are activated to secrete the second wave of cytokines to promote virus clearance (35). If the pro-inflammatory factors in cytokines are overproduced, the pro-inflammatory response will increase significantly, and the anti-inflammatory response will be under controlled, which will become a cytokine storm, which is one of the reasons for the increase in mortality during influenza infection (36). Therefore, influenza treatment is important in the early stages of infection. It can effectively reduce the replication of influenza viruses and avoid the aggravation of lung injury caused by the release of large amounts of inflammatory factors.

TCM has been extensively studied for the treatment of influenza, both as monomers and as preparations of TCM compounds. One study found that TCM compounds can inhibit influenza virus replication and inflammatory cytokines (37). Some studies screened TCM with neuraminidase inhibitory effect by comparing antiviral drugs, and found that *Coptidis Rhizoma*, *Isatidis Folium*, *Lonicerae Flos*, *Scutellaria Radix*, *Cyrtomium Rhizome*, *Houttuynia Cordata*, *Gardeniae Fructus*, and *Chrysanthemi Indici Flos* have strong antiviral effects (38, 39). We found that the components of QQXD contain liquitin, baicalin, wogonoside, platycodin D, baicalein, glycorrhizac acid, wogonin, enolone, phillyrin etc. Some studies have found that baicalin could inhibit virus *in vivo* and *in vitro*, and could inhibit inflammatory response (40–44). A previous study showed that phillyrin could improve the survival rate of influenza A mice, and reduce lung index and pulmonary virus titer (27). A study found that glycyrrhizic acid could improve the survival rate of mice infected with H1N1, reduce the virus titer in lung tissue, and reduce the expression of TNF-α, IL-6, and IL-1β (26). In this study, QXD was found to be effective in treating influenza virus pneumonia.

We found that QQXD improved the survival status of influenza mice. mice in Control were in good spirits, had shiny coat colors, normal activity, no changes in food and water intake, and a natural increase in body weight. After 3 days of infection, symptoms of influenza infection were apparent in the mice. Mice in Model had typical flu symptoms, including loose hair, matte hair, frizz, reduced activity, reduced intake of food and water, convulsions and dyspnea. Mice in QXD had smoother hair, reduced activity, reduced food and water intake, and were lighter than those in Model. Influenza virus infection in mice caused weight loss, and mice treated with QQXD had less weight loss. A half lethal dose of influenza virus was used to make the model of influenza virus pneumonia, and a 50% mortality rate was observed on day 8. We found that QQXD improved survival in influenza mice. This is consistent with the previous findings on effective TCM components, such as baicalin. Early application of QQXD in influenza pneumonia mice has been shown to be effective in inhibiting viral replication and achieving therapeutic aims. Influenza virus pneumonia causes excessive secretion of inflammatory factors, which further aggravates lung tissue damage. The weight of lung tissue in influenza virus pneumonia increases significantly, and QQXD can reduce the lung viral load and lung index. Influenza virus pneumonia causes excessive secretion of inflammatory factors, which further aggravates lung tissue damage. QQXD can reduce the expression of IL-1α, IL-4, IL12(P70), and TNF-α inflammatory factors in influenza mice, and prevent the excessive inflammatory reaction from aggravating lung injury.

For viral infections, probiotics may be able to help the host act as an antiviral (45). Influenza virus mainly adsorbs on respiratory epithelial cells and replicates in the lower respiratory tract through the respiratory tract (46). There seems to be no obvious connection with intestinal flora. However, there is evidence that the gastrointestinal tract and respiratory system as well as their respective microbiomes are interconnected and affected, which is called the gut-lung axis (47). Studies have found that probiotics can reduce the susceptibility to influenza infection, reduce the infiltration of inflammatory cells in the lungs and improve the speed of virus clearance (48). On the contrary, the disturbance of intestinal flora after the use of antibiotics can lead to the disorder of helper T cells in mice infected with influenza virus (49). For the changes in intestinal flora caused by influenza infection in mice, GeGen QinLian decoction can help restore the balance of intestinal flora (50). In this study found that beneficial bacteria such as Lactobacillus decreased significantly, Oscllospira increased, and Bacteroides increased in QQXD when influenza virus infected mice. Alpha diversity analysis showed no significant difference in flora abundance between Control and QQXD and between Model. Beta diversity analysis revealed significant differences in microbial communities among the three groups. The intestinal microorganisms of mice infected with the influenza virus were disordered, with a significant increase in the microbial community, which was different from that of normal mice. Opportunistic pathogenic bacteria such as Burkholderia, Delftia, Prevotella and Butyricoccus have increased in Model. Prevotella has been found that it is related to human infection (51), such as increased abundance in patients with AIDS (52), and may be related to a variety of inflammatory diseases (53) and may promote inflammatory response (54). It was found that there was a difference in gut flora between QQXD and Model. Enrichment analysis of different flora showed significant enrichment in human infectious diseases. This suggests that QQXD may be able to treat infectious diseases such as influenza. Compared to Model, QQXD up-regulates cyanoamino acid metabolism and photosynthesis antenna proteins. This may be a potential mechanism by which QQXD modulates the gut flora to achieve therapeutic effects. We found that influenza virus infection increases the abundance and composition of intestinal flora in mice, possibly due to an increase in pathogenic bacteria, and that QQXD has some regulatory and protective effects on intestinal flora of influenza infected mice.

Several studies have found that influenza virus infection can affect the body’s metabolism. Fatty acid metabolism may be inhibited during H7N9 infection, its level may predict a fatal outcome after H7N9 virus infection (55). It was also found that more than 100 differential metabolites, including purines, pyrimidines, acylcarnitines, fatty acids, amino acids, glucocorticoids, sphingolipids and phospholipids, were captured in the sera, lung tissue and bronchioles of the mice. Many of these metabolites belonged to pulmonary surfactants, indicating influenza virus infection induced aberrations of the pulmonary surfactant system might play an important role in the etiology of respiratory failure and repair (56). Another study found that the differentiation of fatty acid biosynthesis and cholesterol metabolism was different in different strains of cells infected with influenza (57). During influenza infection, the metabolism of SCFAs in the host decreases, the SCFAs are produced by bacterial fermentation of colon dietary fiber, and the concentration in the intestinal cavity is high under physiological conditions (33). Another study further found that activation of short chain fatty acid receptor FFAR2 could prevent bacterial double infection (58). Based on the research findings, SCFAs may be a new choice for the treatment of infection (51). TCM can effectively improve lipid metabolism by increasing SCFAs levels, regulating bile acid metabolism, reducing the production of trimethylamine N-oxide, alleviating the release of inflammatory factors, and altering branched-chain amino acids biosynthesis (59). We found that the intestinal metabolism of mice changed after influenza infection, with a significant reduction in the metabolism of short-chain fatty acids and a relative increase in the metabolism of organic acids and fatty acids, which may be related to differential changes in the intestinal flora. The findings of this study are the same as those of previous studies showing that influenza infection leads to a reduction in short-chain fatty acids, but QQXD does not promote the metabolism of short-chain fatty acids, and it may not be possible to treat influenza virus infection through this mechanism. In this study, different metabolites were found between QQXD and Model, and metabolic pathways were analyzed, including aminoacyl tRNA biosynthesis, nitrogen metabolism, phenylalanine, tyrosine and tryptophan biosynthesis, phenylalanine metabolism, valine, leucine and isoleucine biosynthesis.

Enrichment analysis of intestinal differential flora revealed that QQXD upregulated cyanoamino acid metabolism. The main intestinal differential metabolites are indole-3-carboxyacid, indole-3-carboxaldehyde, isoalloLCA, 4-hydroxybenzoic acid reduced, 3-hydroxyphenylacetic acid, aspartic acid, threonine, homoserine, n-acetyltryptophan, and the enrichment analysis is dominated by nitrogen metabolism, phenylalanine metabolism, valine, leucine, and isoleucine biosynthesis. Through the analysis of different flora and metabolites, we found that QQXD can affect the metabolism of influenza mice by modulating the flora of Paracteroids, Pediococcus, and Clostridium. The main metabolic pathway is cyanoamino acid metabolism, which may be the underlying mechanism of QXD in influenza treatment.

The study was an initial look at whether QQXD could inhibit influenza viruses, but it was not clear whether it affected hemagglutinin or neuraminidase. This study needs to further refine the relationship between influenza-induced inflammatory response and changes in intestinal flora. QQXD has been used in clinical practice, but no multicenter randomized controlled trials have been conducted.

In summary, QQXD can effectively treat influenza virus pneumonia by inhibiting virus replication, inhibiting inflammatory factor storms, and protecting lung tissue from damage. The mechanism of effect of QQXD on influenza viral pneumonia is likely to be related to improved gut microbiome dysbiosis, increased gut carbohydrate metabolism, and up-regulating cyanoamino acid metabolism pathways. This study provides a novel understanding of QQXD for clinical applications in influenza virus pneumonia.

## Data availability statement

The datasets presented in this study can be found in online repositories. The names of the repository/repositories and accession number(s) can be found in the article/Supplementary material.

## Ethics statement

All experimental procedures performed on the animals were conducted under the National Institutes of Health Guidelines on Laboratory Research. The experiment was conducted under the supervision and evaluation of the Ethics Committee of the Experimental Animal Center of the Academy of Military Medical Sciences (permit number: 2020-061).

## Author contributions

XX and QL designed the research. HJ and YG completed experimental quality control. XC, TJ, and YC directed the experiment. RS contacted the experiment. BL, SH, CZ, MZ, YH, HY, and JN performed the experiment and analyzed the data. XX, BL, and SH wrote the manuscript. All authors contributed to the article and approved the submitted version.
