# Supplementary material for: Qin-Qiao-Xiao-Du formula alleviate influenza virus infectious pneumonia through regulation gut microbiota and metabolomics
Source: Front Med (Lausanne). 2022 Oct 14;9:1032127. doi: 10.3389/fmed.2022.1032127 (PMC9614278; doi:10.3389/fmed.2022.1032127)

Supplementary Material

1 Supplementary Data

Among them, 18 major compounds, including liquiritin, baicalin, platycodin D, baicalein, wogonoside, glycyrrhizic acid, and enoxolone, etc. The specific components of QQXD were shown in Supplementary Table 1.

This study found that the richness of the intestinal community in Model was higher than those in Control and QQXD (Supplementary Fig.1A and B). The colonies in Model were far related and the intestinal community was more uniform, which was different from Control and QQXD (Supplementary Fig.1C and D). Control, Model and QQXD did not detect the low proportion of species (Supplementary Fig.1E).

The 16s RNA data for this study can be found in the SRA database (SRA accession: PRJNA868290, https://www.ncbi.nlm.nih.gov/sra).

2 Supplementary Figures and Tables

Supplementary Table 1 The main bioactive compounds in plasma after oral administration of QQXD

No.	T_R（min）	Molecular formula	Identification results	Detection
1	3.99			ND
2	7.71	$C_{27}H_{30}O_{15}$	Isoglycyrrhizin glyoxylation	Y
3	14.01			ND

4	14.70	$C_{21}H_{22}O_9$	Liquiritin	Y
5	14.72 (+)			ND
6	15.22 (+)			ND
7	15.33			ND
8	16.78	$C_{27}H_{26}O_{17}$	Baicalin glyoxylation	Y
9	16.83 (+)			ND
10	17.32	$C_{28}H_{28}O_{17}$	Baicalin glucose aldehyde	Y
11	18.69 (+)			ND
12	18.72	$C_{16}H_{12}O_9S$	Hydroxylation and sulfation of baicalein	Y
13	18.83 (+)	$C_{21}H_{18}O_{11}$	Baicalin	Y
14	19.93 (+)			ND

15	20.32			ND
16	20.46 (+)	$C_{22}H_{20}O_{11}$	Wogonoside	Y
17	20.83			ND
18	21.00 (+)	$C_{21}H_{18}O_{11}$	Baicalin isomer	Y
19	21.05	$C_{57}H_{92}O_{28}$	Platycodin D	Y
20	21.09 (+)	$C_{22}H_{20}O_{11}$	Baicalin isomer	Y
21	23.37	$C_{26}H_{32}O_{11}$	Demethylation of phillyrin	Y
22	23.39 (+)	$C_{15}H_{10}O_5$	Baicalein	Y
23	24.13	$C_{42}H_{62}O_{16}$	Glycyrrhizic acid	Y
24	24.90 (+)	$C_{16}H_{12}O_5$	Wogonin	Y
25	25.25 (+)	$C_{16}H_{12}O_5$	Wogonin isomer	Y
26	25.34	$C_{36}H_{54}O_{10}$	Glycyrrhizic acid loses a molecule of glucuronic acid	Y

27	25.94	$C_{15}H_{10}O_8S$	Sulfation of baicalein	Y
28	27.88	$C_{30}H_{46}O_4$	Enoxolone	Y

Supplementary Fig.1 Alpha diversity index. (A) Chao1 index. (B) Observed species richness. (C) Faith's PD index. (D) Pielou's evenness index. (E) Good's coverage index.***$p < 0.001$

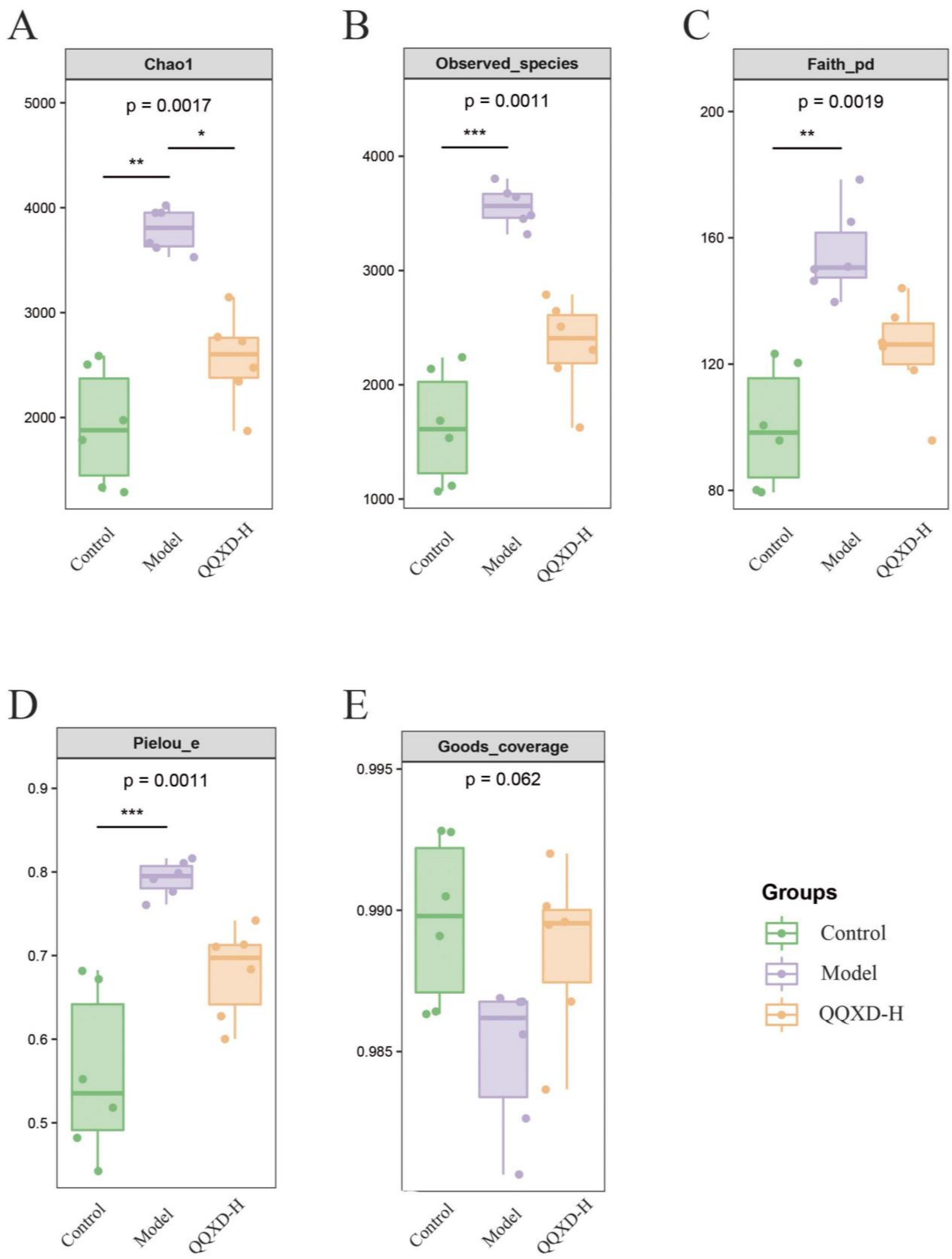

Supplement: Supplementary file 1 [file Data_Sheet_1.pdf]
